# Estimating the Efficacy of a Commercial Phase I Inactivated Vaccine in Decreasing the Prevalence of *Coxiella burnetii* Infection and Shedding in Red Deer (*Cervus elaphus*)

**DOI:** 10.3389/fvets.2017.00208

**Published:** 2017-12-06

**Authors:** David González-Barrio, José Antonio Ortiz, Francisco Ruiz-Fons

**Affiliations:** ^1^Health and Biotechnology (SaBio) Group, Instituto de Investigación en Recursos Cinegéticos IREC (CSIC-UCLM-JCCM), Ciudad Real, Spain; ^2^Medianilla Red Deer Genetics, Benalup-Casas Viejas, Spain

**Keywords:** control, inactivated phase I vaccine, Q fever, wildlife, zoonosis

## Abstract

The red deer (*Cervus elaphus*) is a relevant reservoir for *Coxiella burnetii* in Iberia. *C. burnetii* genotypes that infect red deer also infect humans and domestic animals. Integrated control approaches that target both domestic and wild ruminants are, therefore, required to reduce *C. burnetii* infection risks in Iberia, especially in wildlife–livestock–human interaction scenarios. The aim of this field experiment was to test the efficacy of an inactivated phase I vaccine [Inactivated phase I vaccine (IPIV); Coxevac^®^] when used to control *C. burnetii* shedding prevalence and burden in red deer as a tool to prevent transmission to livestock and humans. A semi-extensively bred red deer population in which *C. burnetii* is endemic was used as a model of the Iberian context. Around 75% of the reproductive hinds (>1 year old; *N* = 441) in the population were first vaccinated early in 2012 and were then revaccinated 3 weeks later; they were subsequently revaccinated biannually until January 2014. 75% of the yearling females left as replacement in 2012 and 2013 were vaccinated in June and revaccinated thereafter following the same protocol. 25% of the population, including the replacement females, was kept as a control group throughout the study. Changes in the humoral immune response after vaccination were estimated by analyzing sera collected at 10 different times between January 2011 and January 2015. The vaccinated and control hinds were surveyed at 2.5, 3.5, and 4.5 months after calving in 2012, 2013, and 2014 to collect vaginal swabs, milk, and feces. The presence and burden of *C. burnetii* DNA in swabs, milk, and feces was evaluated by means of real-time PCR. Vaccination induced high antibody prevalence and levels. The proportion of animals shedding *C. burnetii* in vaginal secretions and milk did not change over time in the vaccination group with respect to the control group. In contrast, there was a significant reduction in the proportion of deer shedding *C. burnetii* in feces in both the vaccinated and control groups. The decrease in the proportion of fecal shedders coincided with a significant reduction in the incidence of infection of non-vaccinated yearling females in the population. This finding suggests that long-term vaccination with IPIV could reduce environmental contamination with *C. burnetii* and control transmission, perhaps making this a promising tool with which to control *C. burnetii* in red deer in the future.

## Introduction

Q fever is a zoonotic disease caused by *Coxiella burnetii*, a Gram-negative bacterium, that infects animals and humans worldwide, causing a high economic impact (1–3); e.g., the total cost of the massive Q fever outbreak in the Netherlands between 2007 and 2010 was estimated as being €600 million (4, 5).

Clinical signs of Q fever in domestic and wild ruminants include returns to estrus, abortions, premature deliveries, stillbirths, and the birth of weak offspring with reduced survival rates (2, 6). It is estimated that Q fever causes around 9% of the abortions in sheep flocks (7), which makes Q fever an important disease in domestic ruminant production. In wildlife, Q fever is a major threat to the success of controlled breeding programs for endangered wild ruminants, e.g., the Saharawi dorcas gazelle (*Gazella dorcas neglecta*) breeding program (Abaigar T., personal communication) and the productivity of farmed wild ungulates (8, 9). *C. burnetii* replicates efficiently in the placenta of ruminants and is subsequently shed in vaginal secretions, milk, and feces by infected females (10). Massive shedding occurs mainly around parturition or after reproductive failure and leads to high concentrations of infective *C. burnetii* in the environment that favors transmission through contaminated aerosols.

Domestic ruminants are the main reservoirs of *C. burnetii* for humans (1). However, *C. burnetii* is a multi-host pathogen with a wide host range (11), and like other multi-host pathogens (12, 13), its transmission at the wildlife–livestock–human interface may have increased with the demographic changes undergone by particular wild species, e.g., ungulates (14). A recent large-scale study proved that the red deer (*Cervus elaphus*) is an important wild reservoir of *C. burnetii* in Iberia (15); 50% of Iberian red deer populations are infected with *C. burnetii*, with an average individual antibody prevalence of 12.2%, which is similar to values reported in domestic ruminant herds (16). Furthermore, red deer females shed *C. burnetii* after infection (9), and specific red deer genotypes infect humans (17). The current increasing demographic trends of red deer populations in Europe (14) would, therefore, ease *C. burnetii* transmission at the red deer–livestock–human interface. Preparedness to fight against Q fever would require integrated approaches targeting both domestic and wild reservoirs. To achieve this, it is necessary to improve current control strategies in domestic ruminants and develop new strategies with which to control the infection in key wild reservoirs in wildlife–livestock–human interaction scenarios.

Q fever control approaches seek to reduce the proportion of *C. burnetii* shedders as a means of reducing environmental contamination and transmission. The most efficient strategy by which to reduce the percentage of shedders in a herd is currently vaccination with inactivated phase I vaccines (IPIVs) (18). The use of IPIV in domestic ruminants reduces the risk of infection of naive animals (19–22). In endemic herds, vaccination with IPIV is less efficient in the short-term, and therefore, long-term vaccination is recommended (19, 23). In this context, this study aims to test the efficiency of the long-term use of commercial IPIV as regards reducing *C. burnetii* shedding in red deer. The efficacy of IPIVs in wild ruminants has not been tested to date; however, epidemiological and clinical studies suggest that *C. burnetii* infection is similar in wild and domestic ruminants (6, 8, 9, 11), and that basic knowledge acquired in domestic ruminants can be applied to wild ruminants. We seek to test the efficiency of IPIV in endemic scenarios because *C. burnetii* infects over 50% of Iberian red deer populations; any laboratory-controlled experimental approach carried out on red deer would most probably mimic what has been previously reported in domestic ruminants. Therefore, we carried out a field longitudinal vaccination experiment and monitored *C. burnetii* shedding prevalence and burden over 3 years using a semi-extensively bred deer population as a model.

## Materials and Methods

### The Study Population

The experiment was performed on a semi-extensively bred red deer population located in the province of Cádiz in southern Spain. The deer are semi-extensively bred in a forest-shrub-prairie habitat divided into different plots by high-wire fencing. The animals are kept in separate batches according to their sex and productive status. The number of deer in the population is around 500 hinds and 100 stags. They are kept within large fenced (6–8 ha) enclosures in batches of 60–80 reproductive females; the males are kept in separate enclosures. The animals are identified with individual ear tags.

A strict management protocol is implemented in the study population so as to minimize the stress associated with handling, especially during critical stages of the production cycle, such as calving. Although bred in controlled conditions, these deer are not domestic animals and consequently get highly stressed when they are restrained for sample collection. The reproductive females (>1 year old) give birth to calves by the end of April. The calves remain with their mothers in enclosures until they are weaned at around 3.5 months of age, after which they are split into male and female batches separated from the hinds and stags. All the calves are handled for routine pathogen monitoring at the age of 2.5, 3.5, and 7 months. The reproductive females are handled annually in winter (January–February) and two/three times in summer (July, August, and September). A selection of 13-month-old yearling females is left annually as replacement, and these are randomly allocated to existing batches of reproductive hinds. The strict handling protocol precluded the design of a more accurate monitoring protocol for the vaccination experiment. Nonetheless, this was still a unique opportunity to test the efficacy of IPIVs in an endemic red deer population, something that is extremely difficult to achieve in a free-roaming deer population. Furthermore, very few attempts to monitor the efficiency of IPIVs in real endemic scenarios have been performed to date and none with wild ruminants.

### Dynamics of *C. burnetii* Infection in the Study Population

The long-term monitoring of *C. burnetii* in the study population confirmed that it is endemic (9, 24, 25). Transmission takes place mainly around the calving season (25). Clinical reproductive signs associated with infection with *C. burnetii* have never been observed directly in this population because the hinds remain undisturbed in enclosures during the last third of the gestation period and around calving. However, *C. burnetii* infection has been associated with increased reproductive failure in the study population (9) and in other deer populations (26).

European wild rabbits (*Oryctolagus cuniculus*) are abundant in the study area and share the habitat with the deer. Rabbits are also true *C. burnetii* reservoirs (27). However, the interference of rabbits in the dynamics of *C. burnetii* infection in deer is expected to be low because distinct *C. burnetii* genotypes infect the deer and rabbits on the study site (17, 28).

### Design of the Experimental Vaccination Trial

An IPIV that has been widely tested on European domestic ruminants was selected for the experiment (Coxevac^®^, CEVA Santè Animale, France). Coxevac is commercially available in Spain, and its use in mammal species is approved by the European Medicines Agency. The experiment was designed in compliance with the recommendations of the vaccine manufacturer. The experiment was approved by the Research Ethics Commission of the Animal Ethics Committee of Castilla—La Mancha University.

The experiment targeted hinds with a history of natural exposure to *C. burnetii* as a means of estimating the usefulness of IPIV as regards reducing the risk of infection in free-ranging infected red deer populations. Previous vaccination experiments carried out on domestic ruminants suggest revaccination every 9–12 months (29, 30). However, long-term series serological data of the study population showed that the average life of antibodies produced after natural infection was around 5–6 months (25). This finding suggested that protection linked to humoral immunity would be boosted with revaccination every 6 months.

**Figure 1:**
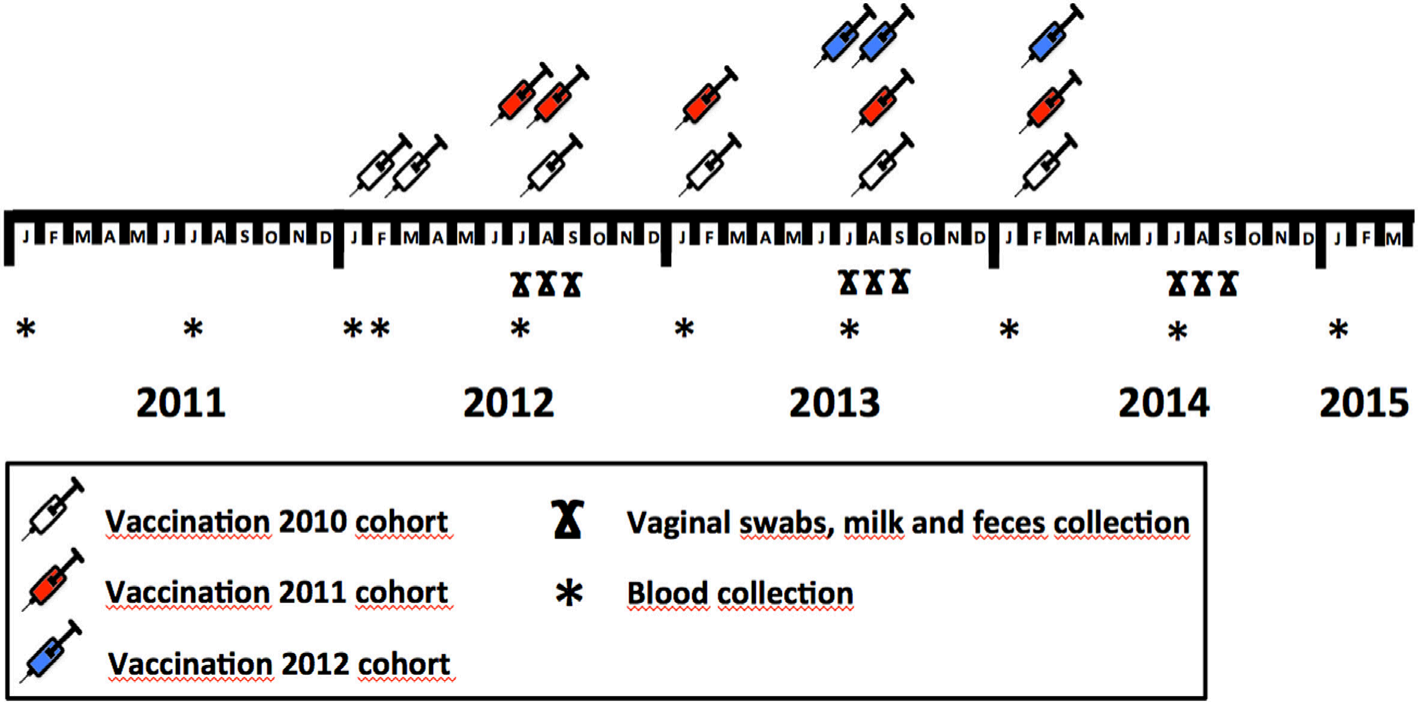
Vaccination and sample collection schedule throughout the study, showing the protocol employed to implement vaccine in different deer cohorts (2010, 2011, and 2012) and sample—blood, vaginal swabs, milk, and feces—collection months within study years.

In January 2012, the first dose of the vaccine was given to 320 of the 441 reproductive hinds comprising the herd (72.6%), while the rest (*n* = 121, 27.4%) were left as a control group (2010 cohort). We allocated vaccinated and control animals to each existing batch on the farm to evaluate the effect of vaccination on coexisting non-vaccinated mates. Any female batch on the farm, therefore, contained both vaccinated and non-vaccinated animals. Vaccinated animals were revaccinated 3 weeks after vaccination (29). Thereafter, the animals in the vaccinated group were revaccinated biannually until January 2014. In June 2012, 93 of the 124 (75.0%) yearling females born in 2011 that were kept as replacement (2011 cohort) were vaccinated. Revaccination was performed 3 weeks later and biannually (Figure 1); 31 animals (25.0%) were left unvaccinated. In June 2013, 104 of the 134 hinds (77.6%) of the cohort of replacement females born in 2012 were vaccinated and were revaccinated 3 weeks later and biannually thereafter; 30 females (22.4%) were left unvaccinated. The proportion of hinds in the vaccination vs. the control group remained at 3:1 throughout the experiment. The vaccine was injected subcutaneously (3 ml dose) with an automatic injector (Serena 5TPFS, Pimex, Spain). A descriptive summary of the vaccination protocol applied to each cohort is provided in Figure 1.

### Monitoring of *C. burnetii* Shedding

In domestic ruminants, Coxevac has shown the potential to reduce the prevalence of shedders and the burden of *C. burnetii* shed (18, 22, 29). In this study, the efficacy of the vaccination was evaluated by collecting vaginal secretions, milk, and feces at different times—2.5 (July), 3.5 (August), and 4.5 (September) months—after calving in 2012, 2013, and 2014 (Table 1; Figure 1). Samples were collected from a random subset of hinds from both the vaccinated and the control groups whenever they were sampled. Vaginal secretions were collected using sterile cotton swabs. The milk was extracted by hand into sterile tubes after disinfecting the deer’s nipples with 1% chlorhexidine and discarding the first three milk shots. Feces were collected directly from the rectum using sterile disposable latex gloves. The vaginal swabs, milk, and feces were transported to the laboratory in a state of refrigeration and preserved frozen at −20°C until their analysis. The researchers took appropriate biosecurity measures during sample collection, transportation, and analysis.

**Table 1:** Sample size throughout sample type and sampling time according to the allocation of animals to vaccinated (Vacc) and control (Cont) groups.

Sample type	Group	Months	Year				
			2011	2012	2013	2014	2015
Vaginal swab	Vacc	July		45	40	62	
		August		17	41	41	
		September		13	28	26	

	Cont	July		25	12	19	
		August		14	10	19	
		September		11	16	5	

	Subtotal			125	147	172	

Milk	Vacc	July		17	26	16	
		August		17	12	34	
		September		18	31	20	

	Cont	July		12	7	6	
		August		13	2	12	
		September		9	17	3	

	Subtotal			86	95	91	

Feces	Vacc	July		13	33	28	
		August		7	36	35	
		September		12	35	25	

	Cont	July		7	11	9	
		August		9	12	11	
		September		12	16	5	

	Subtotal			60	143	113	

Serum	Vacc	January	48	313	77	89	72
		February		313			
		July	80	121	124	87	

	Cont	January	26	120	50	60	57
		February		120			
		July	43	81	89	58	

	Subtotal		197	1068	340	294	129

**Figure 2:**
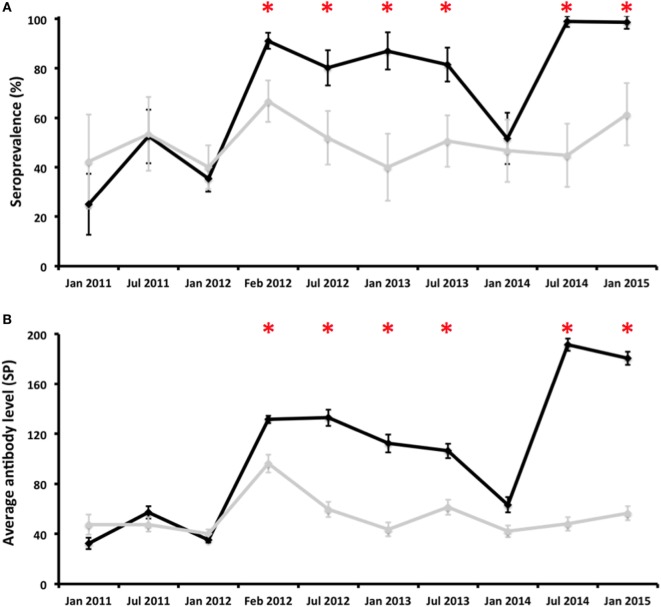
Evolution of the proportion of antibody-positive deer [seroprevalence (%); **(A)**] and average antibody levels [SP; **(B)**] in vaccinated (black line) and control (gray line) groups throughout the experiment. Error bars represent 95% confidence intervals estimated for SER and the estimated SD from the mean for average antibody levels. *Statistically significant differences (*p* < 0.05) between vaccinated and control groups on a particular sampling date.

Moreover, to estimate the presence of *C. burnetii* antibodies in the vaccinated and control hinds before and after vaccination, blood was collected from the jugular vein and placed in sterile 10-ml tubes without an anticoagulant (Table 1; Figure 1). This blood was transported to the laboratory at 4°C, centrifuged at 3,000 *g* for 10 min, and the serum obtained was preserved at −20°C until the analyses were performed. Blood samples were collected at 10 different times between 2010 and 2015 (Figure 2).

### Molecular Analyses

The DNA from vaginal swabs, milk, and feces was extracted using a commercial DNA extraction and purification kit (DNeasy Blood & Tissue kit, Qiagen, Germany) following the protocols provided by the manufacturer. DNA extraction from swabs was optimized by keeping the swabs at 56°C for 30 min in a solution containing 20 µl of proteinase K and 200 µl of AL buffer. The swabs were subsequently vortexed vigorously for 15 s, removed from the tubes, and discarded. The remaining sample was kept for 30 additional minutes at 56°C, after which the manufacturer’s blood extraction protocol was followed. Each sample of milk (200 µl) was mixed directly with ATL and proteinase K and incubated for 3 h at 56°C, and the manufacturer’s blood extraction protocol was then followed. One gram of each fecal sample was mixed with 4 ml of TE buffer (Tris Base 10 mM, EDTA 1 mM, pH 8), vortexed for 30 s, and centrifuged at 3,000 *g* for 2 min. Thereafter, 200 µl of the supernatant were treated with proteinase K (20 µl) and ATL buffer (180 µl) for 30 min at 70°C, and the extraction was completed following the manufacturer’s blood and tissue extraction protocol. The concentration of DNA in each aliquot was quantified (NanoDrop 2000, Thermo Scientific, Waltham, MA, USA), and the aliquots were frozen at −20°C until the PCR was performed. To prevent and detect sample cross-contamination, negative controls (Nuclease free water; Promega, Madison, WI, USA) were included in every 10 samples during the DNA extraction procedure. The DNA samples were analyzed by means of a real-time PCR (qPCR), targeting a transposon-like repetitive region of *C. burnetii* as described previously (15, 31). SsoAdvanced™ Universal Probes Supermix (BioRad, USA) was used in qPCR according to the manufacture’s specifications. DNA extraction and PCR were performed in separate laboratories under biosafety level II conditions (BIO II A Cabinet, Telstar, Spain) to avoid cross-contamination. DNA from Coxevac was used as a qPCR positive control. Samples were considered positive to the presence of *C. burnetii* DNA at a cycle threshold (Ct) below 40.0 (31).

### Serological Analyses

The presence of specific antibodies against *C. burnetii* in deer sera was determined by using a commercial indirect ELISA test (PrioCHECK™ Ruminant Q Fever Ab Plate Kit, Thermo Fisher Scientific, USA), as previously reported (15). The ELISA results were expressed as the sample-to-positive control ratio (SP). For each sample, the SP was calculated according to the formula:

$$\text{SP}=\frac{\left(\text{ODs}-\text{ODnc}\right)}{\left(\text{ODpc}-\text{ODnc}\right)}\times 100,$$

where ODs is the optical density of the sample at a dual wavelength of 450–620 nm, ODnc is the optical density of the negative control, and ODpc is the optical density of the positive control. All SP values ≤40 were considered negative, whereas S/P values >40 were considered positive. The SP ratio was considered as a proxy of the level of antibodies against *C. burnetii*, as suggested by the manufacturer.

### Statistical Analyses

Statistical analyses were carried out to compare the prevalence and shedding burden of *C. burnetii* in vaginal swabs, milk, and feces in vaccinated vs. control groups to test the hypothesis that deer immunized with Coxevac would undergo a reduction in *C. burnetii* shedding. The humoral immune response to vaccination in comparison to the control animals was evaluated by comparing seroprevalence and average antibody levels.

Chi-square tests were employed to compare *C. burnetii* DNA prevalence in vaginal swabs, milk, and feces, and antibody prevalence between the vaccinated and control groups at each sampling time. Mann–Whitney *U* non-parametric tests were run to compare the burden of shed *C. burnetii* (average Ct) in vaginal swabs, milk, and feces and antibody levels (average SP) in serum between the vaccinated and control groups. Finally, to assess the effect of time on *C. burnetii* shedding and to attain the evolution of the level of antibodies in vaccinated and control groups, we performed Spearman correlations.

Statistical analyses were run using IBMS SPSS v22.0 software (IBM, Armonk, New York, NY, USA). Ninety-five percent confidence intervals (95% CIs) were estimated for prevalence values according to the expression 95% CI = 1.96 [*p*(1 − *p*)/*n*]^1/2^, where “*p*” is the prevalence in a unitary value and “*n*” is the size of the sample employed to estimate the prevalence.

## Results

### Humoral Immune Response after Vaccination

Two hundred and two hinds that were vaccinated in January 2012 (2010 cohort) were ELISA negative before vaccination. Three weeks after vaccination, 187 of them (92.6%) were ELISA positive. Five months after revaccination, 170 of them were re-analyzed by means of ELISA, and all of them (100.0%) were positive. The effect of vaccination on the humoral response was also evidenced by the increase in the average level of antibodies; seronegative hinds had an average SP value of 14.4 right before vaccination that increased after vaccination (132.1) and remained similar after revaccination (121.9). In contrast, hinds from the control group that were ELISA negative before the start of the experiment had average SP values of 14.9 in January 2012, 86.5 in February 2012, and 65.7 in July 2012. 90.0% (9/10) of the deer from the 2011 cohort that were ELISA negative before vaccination were seropositive 3 weeks later. The level of antibodies underwent a fivefold increase from June 2012 (21.7) to July 2012 (99.1) in the vaccinated group; the average SP values in the control group remained similar throughout this period: 19.8 and 21.7, respectively. The change in the presence and level of antibodies after vaccination could not be estimated for the 2012 cohort because none of the seronegative individuals surveyed right before vaccination (June 2013) was surveyed in July 2013.

Sera (*N* = 2028) were obtained from experimental hinds at 10 different times from January 2011 to January 2015 (Table 1). Antibody prevalence remained above the 80% in the vaccination group after the first vaccination—with the exception of January 2014—and above that observed in the control group (Figure 2). Differences in antibody prevalence between the vaccination and control groups were statistically significant at different times after vaccination (Figure 2). The pattern observed in the average antibody level was similar (Figure 2). Indeed, the vaccinated animals had average SP values close to 200, an unusual SP value in naturally infected ruminants (16). Differences in antibody levels between the vaccinated and control hinds after vaccination were always statistically significant, except in January 2014 (Figure 2). The seroprevalence and average antibody levels had statistically significantly positive time trends in the group of vaccinated animals (rho = 0.697, *p* < 0.05 and rho = 0.745, *p* < 0.05, respectively); neither the seroprevalence nor the antibody level in the control group had statistically significant time trends (Figure 2).

### Patterns of *C. burnetii* Shedding after Vaccination

A total of 444 vaginal swabs, 272 milk samples and 316 fecal samples from 319 hinds (228 vaccinated and 91 controls) were investigated in 2012, 2013, and 2014 (Table 1). The shedding patterns of *C. burnetii* in vaginal secretions, milk, and feces were analyzed separately.

**Figure 3:**
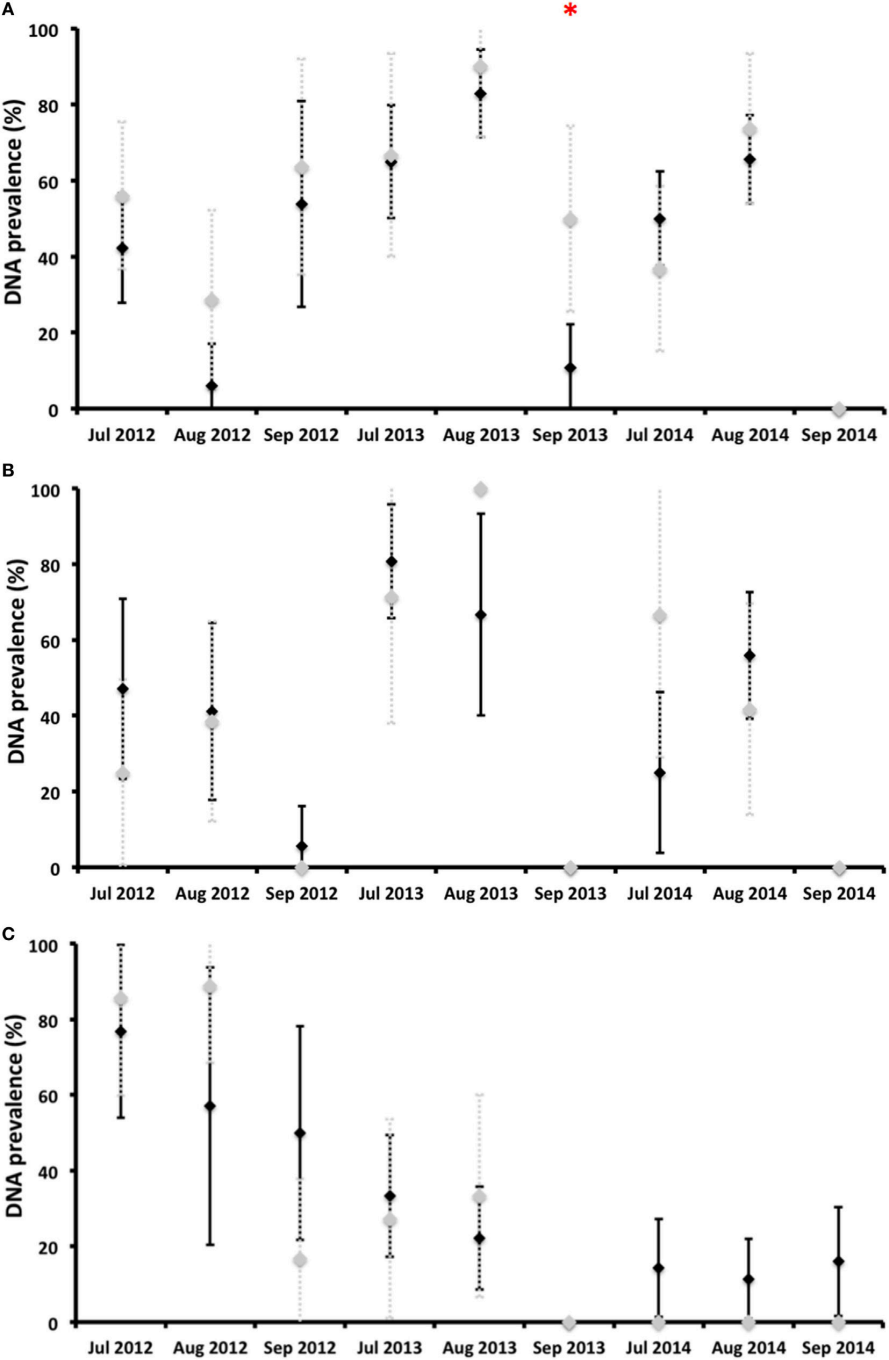
Proportion of deer hinds (prevalence) shedding *Coxiella burnetii* in vaginal secretions **(A)**, milk **(B)**, and feces **(C)** in vaccinated (black diamonds) and control (gray diamonds) groups. *Statistically significant differences (*p* < 0.05) between vaccinated and control groups on a particular sampling date.

**Figure 4:**
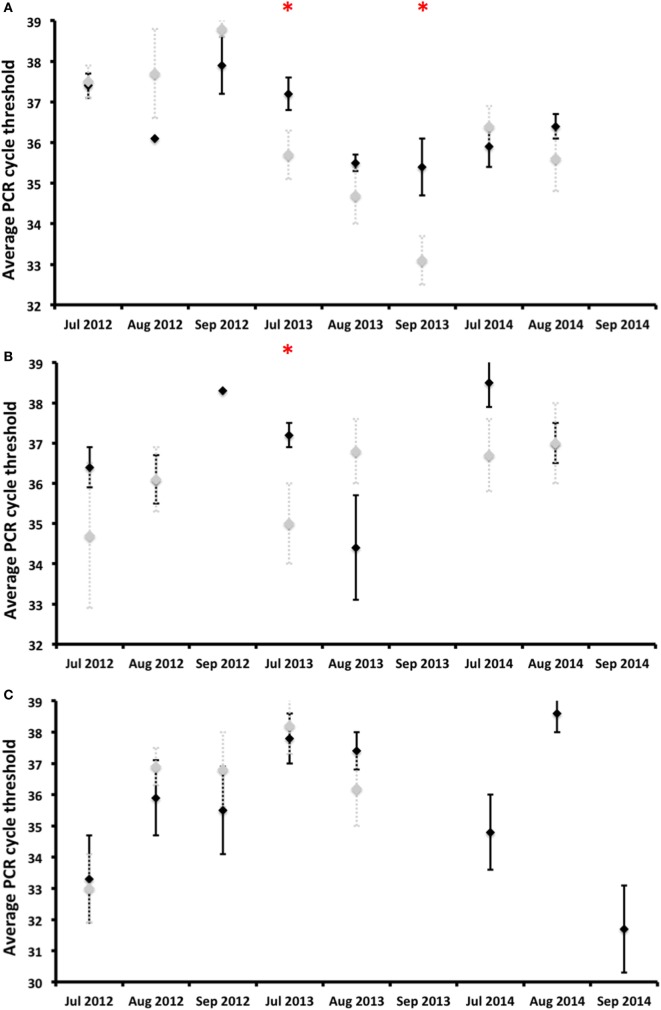
Burden of *Coxiella burnetii* shed [average qPCR cycle threshold (Ct) values] by deer hinds and associated SD (error bars) as regards vaginal secretions **(A)**, milk **(B)**, and feces **(C)** displayed for vaccinated (black diamonds) and control (gray diamonds) groups. *Statistically significant differences (*p* < 0.05) between vaccinated and control groups on a particular date.

*Coxiella burnetii* DNA was detected in vaginal swabs, milk, and feces during any of the surveys carried out after calving, that is, until almost the fifth month after calving (Figure 3). Almost no statistically significant differences in shedding prevalence and in average qPCR Ct values were observed between the vaccinated and control animals (Figures 3 and 4). In general terms, the shedding prevalence in vaginal swabs, milk, and feces (Figure 3), although not the burden of shed bacteria (Figure 4), tended to decrease from the time of calving in any of the three years surveyed and in both the vaccination and control groups. Interestingly, shedding in milk seems to be more limited as regards the time after calving than shedding through vaginal mucus and feces (Figure 3). Another interesting observation was the absence of vaginal shedding in September 2014 and in both the vaccinated and control groups, which contrasts with previous years.

The shedding prevalence of *C. burnetii* in feces, but not the burden of shed bacteria, had a statistically significant decreasing time trend in both the vaccinated (rho = −0,850, *p* < 0.01) and control (rho = −0.870, *p* < 0.01) groups (Figure 3). In 2012, the average shedding prevalence was over 50%, in contrast to 2014 in which it was below 20%, when none of the surveyed animals belonging to the control group shed *C. burnetii* through feces.

## Discussion

Inactivated phase I vaccines have been used in domestic ruminants as a tool to reduce *C. burnetii* shedding by infected animals to limit environmental contamination and thus decrease the risk of infection for naive animals and humans (18, 19, 21). For an optimal vaccine efficacy, populations may fulfill one common pre-requisite, *C. burnetii* shall not be present in the population or it shall only circulate at low prevalence (19, 20). However, since *C. burnetii* is widespread in domestic and wild ruminant populations almost worldwide, there have been attempts to estimate the accuracy of IPIV in controlling infection in endemic scenarios. Some of these studies suggest that IPIV might be useful to control *C. burnetii* infection if long-term vaccination is performed (21). Therefore, and since *C. burnetii* infects around 50% of red deer populations in the Iberian Peninsula (15), we opted to design a long-time vaccination trial. For the first time in scientific literature, this field experiment provides evidence that IPIV may be effective in reducing the shedding of *C. burnetii* by red deer if applied on a long-term basis; additionally, it opens a line of research on *C. burnetii* control in wildlife. The chances of delivering vaccines to wildlife to control relevant pathogens, e.g., rabies virus, classical swine fever virus, or animal tuberculosis (32–34), are increasing because of the progress made in research on efficient oral vaccines and vaccine delivery methods to wildlife (35). Vaccinating wildlife to control pathogens is not, therefore, a utopia. If this to be made possible, it is important to evaluate potential approaches in wildlife, especially with regard to pathogens that are shared with domestic animals and humans.

### Methodological Considerations

This study arose from the need to study strategies with which to control *C. burnetii* infection in a semi-extensively bred red deer population in which Q fever was associated with increased reproductive disorders (9). We designed the experiment by assuming that the basic effects of IPIV would not significantly differ in domestic and wild ruminants. We considered that designing a laboratory-controlled vaccination-challenge experiment for red deer would be (i) unaffordable owing to the logistical constraints and the cost of keeping red deer in BSL3 facilities for a long time period; (ii) unnecessary, because the main features of *C. burnetii* infection in wildlife—infection biology, transmission, pathological findings, clinical signs, shedding patterns, risk factors—do not significantly differ from what is reported in domestic ruminants (11); and (iii) useless as regards estimating the efficiency and potentiality of IPIV to control *C. burnetii* infection in red deer in endemic scenarios. The opportunity to test IPIV in a *C. burnetii* endemic red deer population was unique because access to sampling several times per year was warranted by the handling protocol. The negative aspect of targeting wildlife, although in a controlled population, is that consecutive sampling was not possible, and therefore, any short-term variation in shedding patterns associated with the vaccine may have gone unnoticed.

### Humoral Immune Response to Vaccination

Vaccination and subsequent revaccination induced a high and stable humoral response in the population that remained high when the animals were revaccinated biannually. Vaccination with a single dose (first vaccination) induced a high humoral response in seronegative hinds in a short period of time (3 weeks). Although the boosting effect on the humoral immune response after revaccination could not be evaluated in the short term, the level of antibodies remained similarly high in vaccinated, previously seronegative animals a few months later. This shows that revaccination every 6 months would keep high levels of antibodies circulating in vaccinated individuals. In goats, 95% seroconversion rates were observed 28 days after vaccination, and antibodies lasted 8–12 months (18). Seroconversion rates in vaccinated sheep range between 40 and 100% (21, 36, 37). Brooks et al. (38) detected antibodies until 11 months in sheep vaccinated with a phase I vaccine. 80% of cattle maintain antibodies 1 year after vaccination (30). In contrast, the potential vaccination failure in July 2013 (see below) showed that the average life of vaccine antibodies is shorter than in domestic ruminants.

Although we can conclude that the vaccination and revaccination of deer with Coxevac maintain high levels of anti-*C. burnetii* antibodies circulating, we observed a gap in the response after vaccination in July 2013; seroprevalence and antibody levels decreased by January 2014 (Figure 2). We have no explanation for this observation, but since *C. burnetii* antibodies in red deer last for an average of 5 to 6 months (25), this drop in the humoral response to the vaccine could be related to either the bad conservation of the vaccine or a failure of the vaccine batch employed. We assume that there were no changes in the immune capacity of the animals studied, since significant health problems were not observed in this period. Problems related to the ELISA kit batch employed were also disproved.

### Effects of Vaccination on *C. burnetii* Shedding Patterns

*Coxiella burnetii* DNA was detected in vaginal swabs of vaccinated and unvaccinated hinds 4.5 months after calving, similarly to reports obtained for goats (20). Vaccination did not reduce the burden of *C. burnetii* shed in vaginal secretions throughout the study period, but the shedding time in vaginal secretions was reduced by the third year in comparison to the two previous years. A longer monitoring period would, perhaps, better show the effect of vaccination [see Ref. (29)], which was unfortunately impossible in our experiment owing to logistical and economic constraints. In vaccinated challenged goats, Arricau-Bouvery et al. (18) observed a reduction in the vaginal shedding time just 2 weeks after vaccination. Although not every vaccinated yearling deer of the 2011 and 2012 cohorts had been exposed to *C. burnetii* by the time of vaccination (Figure 2), we did not observe a general reduction in shedding in a short-time period. However, we observed a reduction in the vaginal shedding time, in spite of the fact that the deer were within a highly contaminated environment. We cannot, therefore, discard the hypothesis that the vaccination of unexposed deer under low infection pressure would reduce vaginal shedding in a shorter period [see Ref. (19)]. In contrast to reports obtained for goats (22), no reduction in the *C. burnetii* shedding burden in vaginal secretions was observed in red deer. In naturally infected sheep vaccinated for 3–4 years, the number of shedders and the vaginal bacterial burden shed also decreased only slightly with time (21, 29), which was interestingly more discrete than that which was observed in red deer with similar to higher individual infection rates (25).

*Coxiella burnetii* shedding time in milk from calving was shorter than vaginal shedding, which is consistent with the milk shedding patterns (4–6 weeks) observed in unvaccinated naturally infected goats (18, 39). The vaccination of goats reduced the bacterial load and time of shedding in milk (22). The shorter time of milk shedding, when compared to that of vaginal secretions or feces, coincides with previous reports concerning sheep and goats (39–41). The lack of reduction of shedding prevalence and burden coincides with some studies carried out on sheep and goats (29, 39–41).

*Coxiella burnetii* shedding in feces was detected 4.5 months after calving, as previously observed in non-vaccinated goats (39). The main finding of our study was the progressive reduction of *C. burnetii* shedding prevalence in feces, although not the burden of shed bacteria, over time from the implementation of the vaccination. This was observed in both the vaccinated and control groups but, since the animals in both groups were mixed in existing batches, the reduction observed in the control animals could be a consequence of the lower burden shed by vaccinated hinds. This would theoretically reduce environmental contamination—if we assume that feces are the main source of this contamination (42), which would account for the reduced infection pressure. One interesting observation as regards the study population is that the annual incidence of *C. burnetii* infection in yearlings decreased from 2012 to 2014 (25), which could perhaps support the supposition that vaccination with IPIV reduces the risk of infection by *C. burnetii* in red deer. Future attempts might help identify the causality relationships between fecal shedding and infection pressure.

## Conclusion

The objective of this article is to test the efficacy of IPIV in endemic red deer populations that resemble real scenarios and estimate the usefulness of IPIV to control *C. burnetii* infection in free-roaming endemic red deer populations. Therefore, we followed the recommendations for endemic scenarios and used a long-term vaccination approach for the experiment. Our approach showed that IPIV could be effective in reducing *C. burnetii* shedding, especially in feces, if they are applied on a long-term basis (more than 3 consecutive years) and, therefore, account for a reduction in infection pressure. We vaccinated replacement hinds for the first time when they were 13 months old on the basis of preliminary data on *C. burnetii* infection dynamics (25), but we would recommend that future approaches target 5- to 7-month-old animals. That would perhaps protect calves from infection between the loss of maternally derived antibodies (25) and the next calving season when *C. burnetii* is shed by reproducing hinds.

## Ethics Statement

The experiment was approved by the Research Ethics Commission of the Animal Ethics Committee of Castilla—La Mancha University.

## Author Contributions

FR-F and DG-B designed the study, analyzed the data, and wrote the manuscript; DG-B and JO collected samples; DG-B performed molecular and serological analyses; JO critically reviewed the manuscript; and all the authors approved the submitted version of the manuscript.

## Conflict of Interest Statement

The authors declare that the research was conducted in the absence of any commercial or financial relationships that could be construed as a potential conflict of interest.
